# First person – Alec Nickolls

**DOI:** 10.1242/dmm.045724

**Published:** 2020-06-26

## Abstract

First Person is a series of interviews with the first authors of a selection of papers published in Disease Models & Mechanisms, helping early-career researchers promote themselves alongside their papers. Alec Nickolls is first author on ‘Human embryoid bodies as a 3D tissue model of the extracellular matrix and α-dystroglycanopathies’, published in DMM. Alec conducted the research described in this article while a PhD student in Carsten Bönnemann's lab at National Institute of Neurological Disorders and Stroke, National Institutes of Health, Bethesda, MD, USA. He is now a postdoctoral fellow in the lab of Alexander Chesler at National Center for Complementary and Integrative Health, National Institutes of Health, Bethesda, MD, USA, investigating engineered cellular systems for disease modeling and drug discovery.


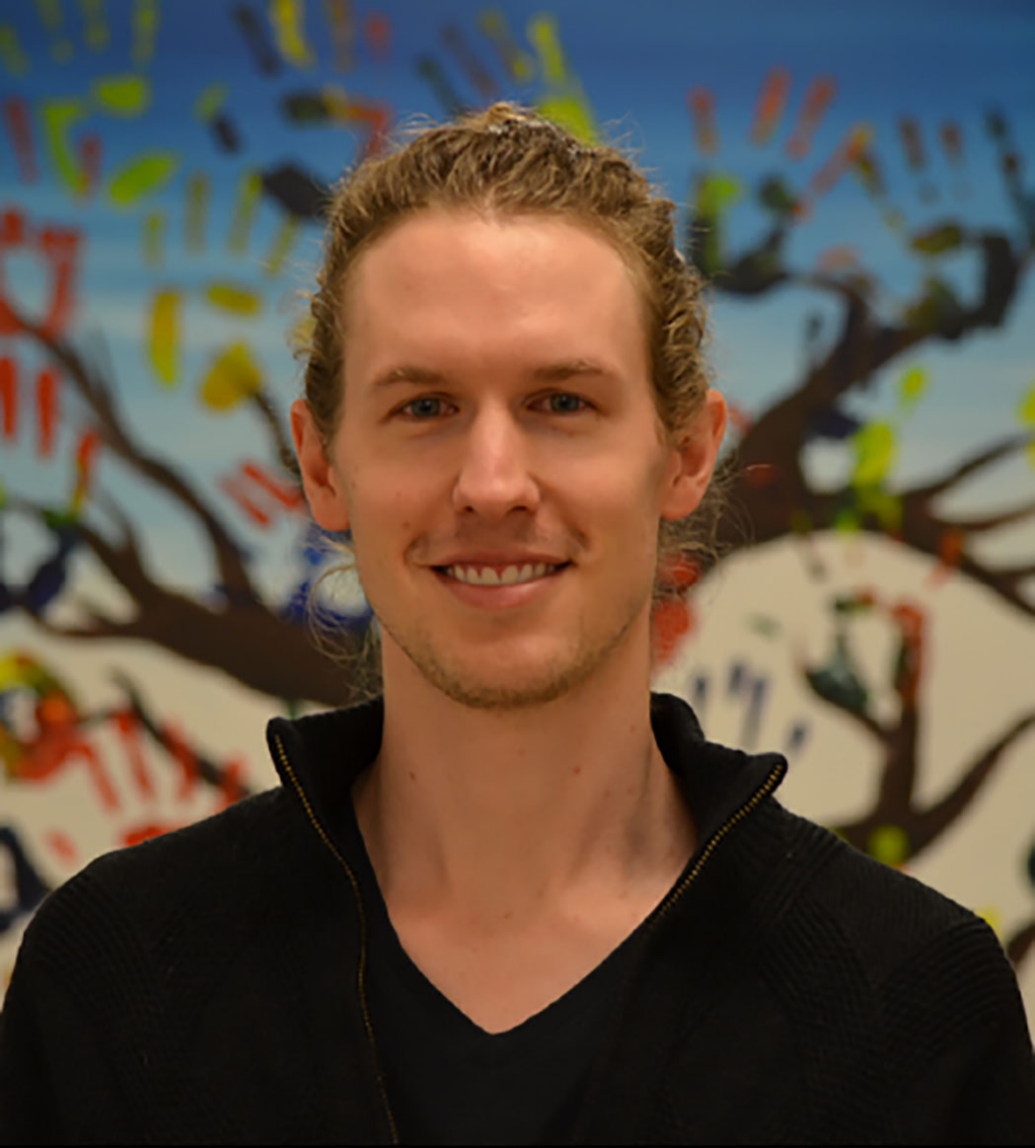


**Alec Nickolls**

**How would you explain the main findings of your paper to non-scientific family and friends?**

The extracellular matrix (ECM) is the microscopic scaffold that supports all our body's tissues. To date, a lot of research has used mice or other animals to study the ECM, because of the difficulty of acquiring human tissue for research. To investigate the ECM in humans, we came up with a method using human stem cells to grow 3D tissue that contains ECM, which we call embryoid bodies. As a proof-of-principle for this approach, we created embryoid bodies in the lab from patients with a condition called α-dystroglycanopathy. The α-dystroglycanopathies are a rare group of diseases – caused by genetic mutations that affect a protein in the ECM called α-dystroglycan – which can lead to malformation of the brain and deterioration of the muscles. Studying embryoid bodies from α-dystroglycanopathy patients has now helped us understand more about their condition; it enabled us to find physical defects in the ECM and to test an experimental therapy for the α-dystroglycanopathies.

**What are the potential implications of these results for your field of research?**

Our study presents a way to investigate the ECM in healthy and diseased human tissue. In the α-dystroglycanopathy field, there are many excellent animal models (over 40 in total) that recapitulate specific forms of the disease spectrum. By generating 3D ECM-containing tissue from individual human subjects, our results add a patient-specific approach to investigating diseases of the ECM such as the α-dystroglycanopathies. Future work that performs parallel testing in available animal models and human tissue models like ours should enable better understanding of relevant disease phenotypes and allow more accurate prediction of drug efficacy in the pre-clinical stage.

**What are the main advantages and drawbacks of the model system you have used as it relates to the disease you are investigating?**

A major disadvantage of using *in vitro* cellular models like ours is that they do not capture the organismal complexity of an animal model – they generally lack mature tissue types, multiple organ systems, mechanical forces, and the typical aging processes that come with being a living, breathing animal. However, *in vitro* approaches also bring specific advantages: greater accessibility for experimental observation and manipulation, shorter experiment duration, potential for high-throughput experiments, and (in the case of our stem cell-based system) a renewable source of patient-specific tissue that enables personalized phenotyping and drug screening.

**What has surprised you the most while conducting your research?**

Several technical challenges – which had put this study on hold – were unexpectedly solved by solutions that came from working on a completely different project. Taking on multiple projects in parallel can be very rewarding; by investing in diverse skillsets, expertise gained from one project can sometimes be applied to another.

**Describe what you think is the most significant challenge impacting your research at this time and how will this be addressed over the next 10 years?**

*In vitro* stem cell-derived tissue currently lacks specificity in terms of the cell types generated, organ-level complexity in terms of physical self-organization and aging-associated biological processes. Many research groups are actively working to address these points, for example, by harnessing specific transcription factors and biochemical signals for more accurate stem cell differentiation, using specialized ECM scaffolds and 3D bioprinting to create complex organoids, and artificially aging cells via genetic and chemical strategies to study age-related diseases. Overcoming the technical hurdles of stem cell-derived tissue specificity and maturation will enable realistic *in vitro* models that more closely recapitulate human anatomy and physiology.

**Figure:**
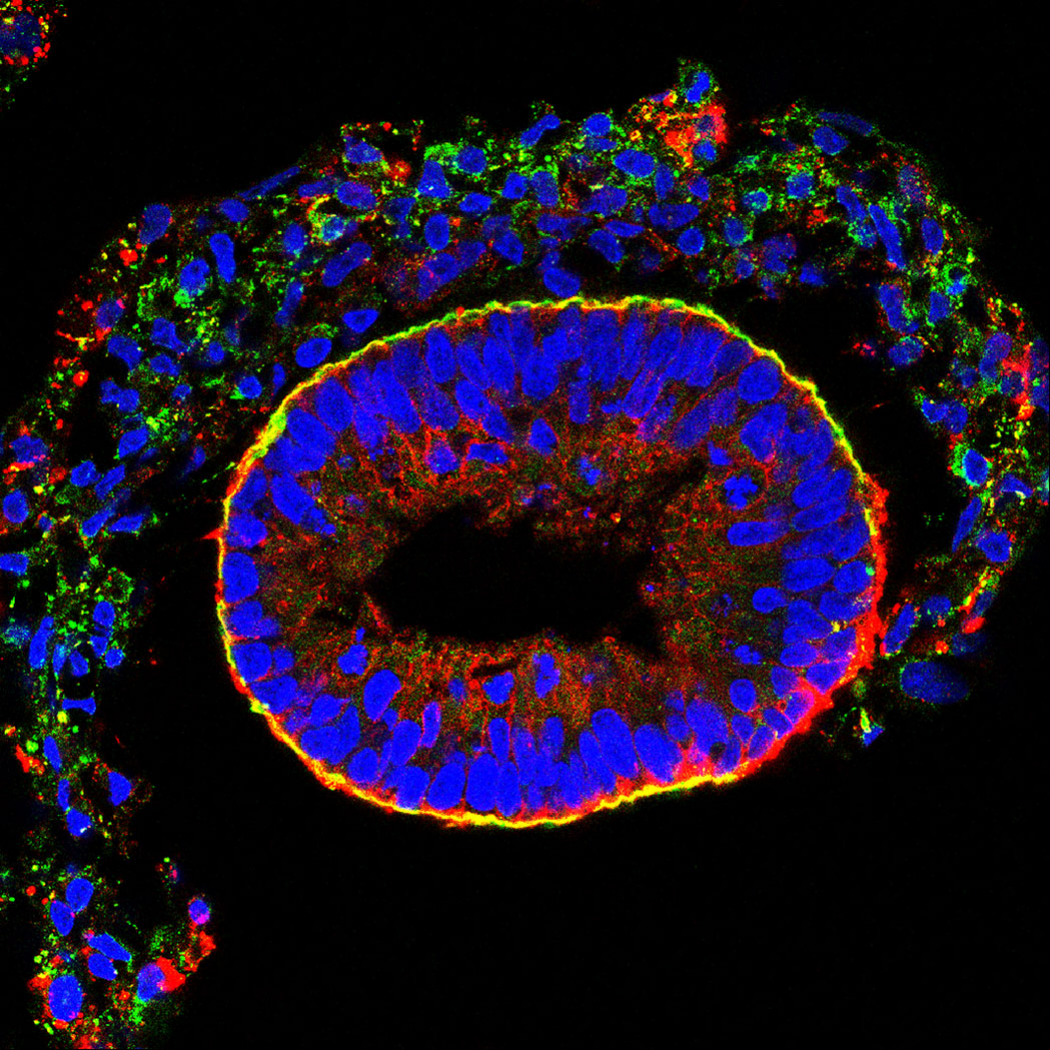
Cross-section of a human embryoid body highlighting one of the main ECM constituents, laminin (green), and its receptor, α-dystroglycan (red) and cell nuclei (blue).

**What changes do you think could improve the professional lives of early-career scientists?**

I think a networking mindset is valuable to scientists at all levels, but it is especially important for early-career scientists. Participating in programs that connect researchers across departments and scientific backgrounds is a great way to find collaborators or even jobs. These opportunities could be especially impactful in shaping someone's career path. One of our lab's most serendipitous collaborations came by attending an intramural core facility presentation – we met an investigator from another institute whose basic research background combined perfectly with our group's clinical expertise to complete several translational projects between the two labs.

**What's next for you?**

I will be carrying out a postdoc fellowship at the National Center for Complementary and Integrative Health, USA, where I will focus on using *in vitro* models for pain research and therapeutics development.
